# DNA hypermethylation and decreased mRNA expression of *MAL, PRIMA1, PTGDR* and *SFRP1* in colorectal adenoma and cancer

**DOI:** 10.1186/s12885-015-1687-x

**Published:** 2015-10-19

**Authors:** Alexandra Kalmár, Bálint Péterfia, Péter Hollósi, Orsolya Galamb, Sándor Spisák, Barnabás Wichmann, András Bodor, Kinga Tóth, Árpád V. Patai, Gábor Valcz, Zsófia Brigitta Nagy, Vivien Kubák, Zsolt Tulassay, Ilona Kovalszky, Béla Molnár

**Affiliations:** 1 2nd Department of Internal Medicine, Semmelweis University, Budapest, Hungary; 2 Molecular Medicine Research Group, Hungarian Academy of Sciences, Budapest, Hungary; 3 1st Department of Pathology and Experimental Cancer Research, Semmelweis University, Budapest, Hungary; 4 Tumour Progression Research Group, Hungarian Academy of Sciences, Budapest, Hungary; 5 Department of Physics of Complex Systems, Eötvös Loránd University, Budapest, Hungary

**Keywords:** DNA methylation, Colorectal cancer, Gene expression, Pyrosequencing, Laser capture microdissection

## Abstract

**Background:**

Colorectal cancer (CRC) development is accompanied by changes in expression for several genes; but the details of the underlying regulatory procesess remain unknown. Our aims were to assess the role of epigenetic processes in tumour formation and to identify characteristic DNA methylation and miRNA alterations in the colorectal adenoma-carcinoma sequence.

**Methods:**

Whole genome expression profiling was performed on colonic biopsy samples (49 healthy normal, 49 colorectal adenoma (AD), 49 CRC); on laser capture microdissected (LCM) epithelial and stromal cells from 6 CRC-normal adjacent tissue (NAT) samples pairs, and on demethylated human CRC cell lines using HGU133 Plus 2.0 microarrays (Affymetrix). Methylation status of genes with gradually altering expression along the AD-CRC sequence was further analysed on 10–10 macrodissected and 5–5 LCM samples from healthy colon, from adenoma and from CRC biopsy samples using bisulfite-sequencing PCR (BS-PCR) followed by pyrosequencing. *In silico* miRNA prediction for the selected genes was performed with miRWALK algorithm, miRNA expression was analysed on 3 CRC-NAT sample pairs and 3 adenoma tissue samples using the Human Panel I + II (Exiqon). SFRP1 immunohistochemistry experiments were performed.

**Results:**

A set of transcripts (18 genes including *MAL, SFRP1, SULT1A1, PRIMA1, PTGDR*) showed decreasing expression (*p* < 0.01) in the biopsy samples along the adenoma-carcinoma sequence. Three of those (*COL1A2, SFRP2, SOCS3*) showed hypermethylation and *THBS2* showed hypomethylation both in AD and in CRC samples compared to NAT, while *BCL2, PRIMA1* and *PTGDR* showed hypermethylation only in the CRC group. miR-21 was found to be significantly (*p* < 0.01) upregulated in adenoma and tumour samples compared to the healthy colonic tissue controls and could explain the altered expression of genes for which DNA methylation changes do not appear to play role (e.g. *BCL2, MAL, PTGS2*). Demethylation treatment could upregulate gene expression of genes that were found to be hypermethylated in human CRC tissue samples. Decreasing protein levels of SFRP1 was also observed along the adenoma-carcinoma sequence.

**Conclusion:**

Hypermethylation of the selected markers (*MAL, PRIMA1, PTGDR* and *SFRP1*) can result in reduced gene expression and may contribute to the formation of colorectal cancer.

**Electronic supplementary material:**

The online version of this article (doi:10.1186/s12885-015-1687-x) contains supplementary material, which is available to authorized users.

## Background

Colorectal cancer (CRC) is regarded as one of the most frequent malignant tumours globally [1]. This heterogeneous disease can develop through at least three distinct molecular pathways by which genetic and/or epigenetic dysregulation influences gene expression and protein levels finally leading to colorectal adenoma and carcinoma formation [2, 3]. One of the epigenetic alterations that can contribute to CRC formation is the abnormal DNA hypermethylation of promoters, resulting in reduced or absent gene expression [4]. DNA hypermethylation occurs at regulatory sites e.g. promoters in a tissue- and cancer type-specific manner [5]. Besides genetic alterations, DNA hypermethylation of tumour suppressor genes is a frequently detected mechanism behind the inactivation of these genes leading to tumour initiation [6]. Although more and more genes are associated with various types of cancers, our knowledge of DNA methylation markers in CRC development remains incomplete.

Another key posttrancriptional epigenetic regulator of gene expression, miRNA, regulates the stability and translation process of mRNAs. The expression of miRNAs has been shown to differ in colorectal tumours compared to healthy colon tissue specimens and on the basis of several experimental results they play role in colorectal cancer formation. Up- and downregulation of certain miRNAs was identified along the adenoma-carcinoma sequence of CRC and evidence supports the role of miRNAs in CRC development and progression as these small non-coding RNAs affect proliferation and invasion [7].

The identification of genes affected by epigenetic changes can be achieved using whole genome gene expression analysis [8]. DNA methylation and miRNA expression alterations can both lead to a certain degree of dowregulation of mRNA expression and consequently of protein levels, which can be confirmed by immunohistochemistry.

In the present study, our aims were (1) to identify DNA methylation markers in CRC samples on the basis of whole genome gene expression analysis and (2) to analyse the DNA methylation levels of these candidate marker along the colorectal adenoma-carcinoma sequence on colorectal adenoma and cancer samples. Furthermore, (3) our aim was to confirm the relationship between gene expression, DNA methylation status, miRNA expression and protein levels of the analysed candidate markers.

## Methods

### Selection of candidate genes regulated by DNA methylation

The selection of candidate genes was based on expression data generated from 147 colonic biopsy specimens (from 49 normal, 49 adenoma, and 49 CRC patients), laser capture microdissected colonic epithelial cells (from 6 NAT, 6 adenomas, and 6 CRC), analysed in a previous study by whole genome HGU133 Plus 2.0 microarrays (Affymetrix) [8, 9]. These data files are available in the Gene Expession Omnibus database (http://www.ncbi.nlm.nih.gov/geo/) at GSE series accession numbers GSE4183 (8 normal, 15 adenoma and 15 CRC), GSE10714 (3 normal, 5 adenoma and 7 CRC), GSE37364 (38 normal, 29 adenoma and 27 CRC)) and GSE15960 (laser microdissected colonic epithelial cells from 6 normal, 6 adenoma and 6 CRC).Clinical data of patients involved in the analysed gene expression studies can be found in Additional file 1: Table S1. 

Although the bioinformatic analysis and the candidate selection was based on previously performed and published raw gene expression data of HGU133 Plus 2.0 microarrays, the aim of the present study was substantially different from the previously published studies’. We aimed to identify genes with gradually altering expression in adenoma and tumour samples that can be potentially regulated by DNA methylation. The data sets GSE4183 [10], GSE10714 [11], GSE 37364 [9], and GSE15960 [8] were analysed to identify genes potentially regulated by DNA methylation. Transcripts with gradually decreasing or increasing expression along the adenoma-carcinoma sequence were selected on the basis of Kendall (tau coefficient) rank correlation analysis (−0.5 ≤ tau coefficient ≤ 0.5). DNA methylation analysis was performed for genes with CpG island(s) on the basis of *in silico* prediction by the CpG Plot EMBOSS application (http://www.ebi.ac.uk/Tools/seqstats/emboss_cpgplot/) [12].

Expression of the selected gene set was also analysed on gene expression data sets of human colorectal cell lines before and after DNA demethylation treatment with 5-Aza (GSE29060: 10 μM 5-Aza treatment for 72 h on HT-29 cell line; GSE14526: 3 μM 5-Aza treatment for 72 h on HCT116 and SW480 cell lines; GSE32323: 0.5 μM 5-Aza treatment for 72 h on Colo32, HCT116, HT-29, RKO and SW480 cell lines.

Student's *t* -test and Benjamini-Hochberg method were applied in order to determine significance of gene expression and DNA methylation level comparisons (*p* < 0.05). For logFc, abs (differences of average of intensity values) > 1 threshold was applied.

### Tissue sample collection

For DNA methylation analysis, tissue specimens were obtained from surgically removed colon tumours (moderately differentiated, Dukes B-C stages; MSS) (*n* = 15) and from histologically normal adjacent tissue (NAT) (*n* = 15) derived from the furthest available area away from the tumour. In addition, adenomas (*n* = 15) were also analysed, containing biopsy samples (*n* = 10) and fresh frozen tissue samples (*n* = 5), as well. Fresh frozen samples were snapfrozen in liquid nitrogen directly after surgery and were stored at −80 °C. Written informed consent was obtained from all patients; and the study was approved by the local ethics committee (Ethics Committee approval was obtained Nr.: TUKEB 2005/037 and TUKEB Nr.: 2008/69, Semmelweis University Regional and Institutional Committee of Science and Research Ethics, Budapest, Hungary). The study was performed according to the ethical standards of the revised version of Helsinki Declaration. Clinical data of patients involved in the study can be found in Additional file 2: Table S2.

### Laser capture microdissection, macrodissection

Frozen tissue samples were embedded in OCT compound (Sakura Finetek, Japan). Then, 10 μm cryosections were cut at −20 °C in a cryostat instrument and mounted on PALM Membrane Slides 1.0 PEN (Carl Zeiss, Bernried, Germany). After fixation with 70 % ethanol for 5 min and absolute ethanol for 2 min, slides were stained with cresyl violet acetate (Sigma-Aldrich, St. Louis, USA). Colonic epithelial and stromal cells (approx. 10^3^ cells) were collected using the PALM Microbeam laser capture microdissection system (PALM, Bernried, Germany). Macrodissected samples were collected from cryosections after toluidine blue staining. Selected areas containing both stromal and epithelial cells were harvested by scratching the tissue slide with a single-use needle.

### DNA methylation analysis

#### Bisulfite conversion

Bisulfite conversion was performed using the EZ DNA Methylation Direct Kit (Zymo Research) without prior DNA isolation. Proteinase K digestion was performed in 20 μl (according to Section I Protocol A) followed by bisulfite conversion. The elution volume was 20 μl.

#### Bisulfite-specific PCR (BS-PCR)

*In silico* CpG island prediction was performed by CpG Plot EMBOSS Application (http://www.ebi.ac.uk/Tools/seqstats/emboss_cpgplot/). Bisulfite-specific PCR reactions were performed using primers designed with PyroMark Assay Design software (SW 2.0, Qiagen, Hilden, Germany) to be specific for non-CpG regions in order to amplify the bisulfite converted DNA samples without discriminating between methylated and non-methylated sequences (Table 1). PCR primers in the opposite direction of sequencing primers were biotin labelled. Primer specificities were tested *in silico* by BiSearch software (http://bisearch.enzim.hu) [13].

**Table 1:** Genes analysed in the study. Genes with gradually decreasing or increasing expression along the adenoma-carcinoma sequence with predictable CpG islands were selected on the basis of Kendall (tau coefficient) rank correlation analysis (−0.5 ≤ tau coefficient ≤ 0.5)

Gene symbol	Gene name
ALDH1A3	aldehyde dehydrogenase 1 family, member A3
BCL2	B-cell CLL/lymphoma 2
CDX1	caudal type homeobox 1
COL1A2	collagen, type I, alpha 2
CYP27B1	cytochrome P450, family 27, subfamily B, polypeptide 1
ENTPD5	ectonucleoside triphosphate diphosphohydrolase 5
FADS1	fatty acid desaturase 1
MAL	mal, T-cell differentiation protein
PRIMA1	proline rich membrane anchor 1
PTGDR	prostaglandin D2 receptor (DP)
PTGS2	prostaglandin-endoperoxide synthase 2
SFRP1	secreted frizzled-related protein 1
SFRP2	secreted frizzled-related protein 2
SOCS3	suppressor of cytokine signaling 3
SULF1	sulfatase 1
SULT1A1	sulfotransferase family, cytosolic, 1A, phenol-preferring, member 1
THBS2	thrombospondin 2
TIMP1	metallopeptidase inhibitor 1

BS-PCR reactions were performed using AmpliTaq Gold 360 mastermix (2x) (Life Technologies, Carlsbad, USA), LightCycler 480 ResoLight Dye (40x) (Roche Applied Science), primer mix (200 nM final concentration), bisulfite converted DNA samples (approx. 10 ng bcDNA/well) in 15 μl final volume. Real-time PCR amplification was carried out with the following thermocycling conditions on the LightCycler 480 System: 95 °C for 10 min, then 95 °C for 30 s, 60 °C with a 0.4 °C decrease/cycle for 30 s, 72 °C for 30 s for 10 touchdown cycles, followed by the amplification at 95 °C for 30 s, 56 °C for 30 s, and 72 °C for 30 s in 40 cycles.

Providing single-base resolution information about the methylation status of a CpG island direct sequencing is one of the most robust methods to analyse BS-PCR products. After bisulfite treatment and BS-PCR, all cytosines are converted to thymines except for those originally methylated. Two different pyrosequencing technologies were applied to analyse DNA methylation of BS-PCR products i.e. the Qiagen PyroMark System and the Roche GS Junior System utilising the 454 technology. The read length of the different technologies differs. With the PyroMark system sequences, up to 60 bp can be analysed, while up to 400 bp read length could be achieved with the 454 technology.

#### PyroMark Q24 sequencing

Pyrosequencing was performed on a PyroMark Q24 instrument (Qiagen) using PyroMark Gold Q24 Reagents (Qiagen) according to the manufacturer’s recommendations. Purification and subsequent processing of the biotinylated single-stranded DNA were performed in two consecutive runs by applying two different sequencing primers in order to cover more CpG sites in the amplicons [14, 15]. Sequencing results were analysed using the PyroMark Q24 software v2.0.6 (Qiagen).

#### GS Junior sequencing

Library preparation with ligated adaptors and emulsion-PCR amplification were as described in “Guidelines for Amplicon Experimental Design”. The concentrations of BS-PCR amplicons were measured by Qubit fluorometer with High Sensitivity dsDNA reagent (Life Technologies). Amplicons belonging to the same sample were pooled at an equimolar ratio and PCR products were purified with AMPure beads (Agencourt, Beckman Coulter Genomics, Pasadena, USA) according to the manufacturer’s standard protocol. The Agilent Bioanalyzer was used with the High Sensitivity DNA Chip (Agilent, Santa Clara, USA) to assess sample quality. Fragment End Repair was performed using the GS FLX Titanium Rapid Library Preparation Kit (Rapid Library Preparation Method Manual 3.2). RL MID Adaptor Ligation was carried out using GS FLX Titanium Rapid Library Preparation Kit (Rapid Library Preparation Method Manual 3.4). After ligation, purification of amplicon libraries was performed with AMPure beads, and assessment of library quality was done using the Agilent Bioanalyzer with High Sensitivity DNA Chip. Library quantification was performed based on fluorometric measurements with Qubit High Sensitivity dsDNA reagent. Equimolar mixing of the libraries was performed by MIDs identifying different samples with different MID adaptors. Amplicon library pools were then amplified by emPCR at a 0.5 DNA molecule per bead ratio using the Lib-L emPCR Kit. Since amplicon lengths were short, the emPCR procedure was performed with reduced Amp Primer quantity (emPCR Amplification Method Manual – Lib-L, GS Junior Titanium Series, Live Amp Mix for paired end libraries). Bead enrichment and sequencing were performed using the GS Junior Titanium Sequencing Kit and the method described in the Sequencing Method Manual, GS FLX Titanium Series.

The Smith-Waterman algorithm with Gotoh’s improvement was used for matching the reads to template sequences in the JAligner software package [16, 17]. As 454 technology can result in sequencing errors with homopolymer stretches e.g. in bisulfite-sequencing templates [18], gaps or insertions were frequently observed in the sequenced reads. Reads with a minimum of 80 % of maximum alignment score were analysed further, after which the actual nucleotides at the potential methylation sites were summarised.

### miRNA analysis

miRNA analysis was performed on an independent formalin-fixed, paraffin-embedded (FFPE) sample set including CRC (*n* = 3), adenomas (*n* = 3) and NAT (*n* = 3) samples. miRNA isolation was performed with the High Pure miRNA kit (Roche) and the expression of approximate 800 miRNA were assessed on Human Panel I + II (Exiqon) with the miRCURY^TM^ Universal RT microRNA PCR protocol according to the manufacturer’s instructions. Normalisation of raw Ct data was performed with interplate calibrators followed by miR-423-5p, as a housekeeping gene expressed at relatively constant levels in our analysed samples. *In silico* miRNA prediction was performed for all analysed genes using the miRWALK database prediction algorithm including validated mRNA targets [19] in order to select experimentally verified miRNA interaction information associated with genes, pathways, organs, diseases, cell lines, OMIM disorders, and literature on miRNAs. Subsequently, expression of selected miRNAs in normal, adenoma and cancer samples was compared.

### Immunohistochemistry

Among the analysed 18 genes, SFRP1 protein level was analysed because of the special interest of our working group. Surgically removed colonic tissues from NAT (*n* = 10), AD (*n* = 10), and CRC specimens (*n* = 10) were fixed in formalin and embedded in paraffin and tissue microarrays (TMA) were constructed. Four μm sections were cut, deparaffinised, and rehydrated. For SFRP1 staining, antigen retrieval was performed in TRIS EDTA buffer (pH 9.0) using a microwave (900 W for 10 min, 340 W for 40 min). Samples were incubated with anti–SFRP1 rabbit polyclonal antibody (ab4193, Abcam, Cambridge, UK) diluted 1:800 for 60 min at 37 °C. EnVision + HRP system (Labeled Polymer Anti-Mouse, K4001, Dako) and diaminobenzidine-hydrogen peroxidase–chromogen substrate system (Cytomation Liquid DAB + Substrate Chromogen System, K3468, Dako) were used with hematoxylin counterstaining. Slides were digitalised using the Pannoramic Scanner p250 Flash instrument (software version 1.11.25.0, 3DHISTECH Ltd., Budapest, Hungary), and analysed with a digital microscope software (Pannoramic Viewer, v. 1.11.43.0. 3DHISTECH Ltd., Budapest, Hungary). The semiquantitative Quick-score (Q) method was applied for SFRP1 protein level alteration analysis. Every TMA core was scored by multiplying the percentage of positive cells by the given intensity value (0 for no staining, +1 for weak, +2 for moderate, and +3 for strong diffuse immunostaining).

## Results

### Gene expression analysis

**Fig. 1:**
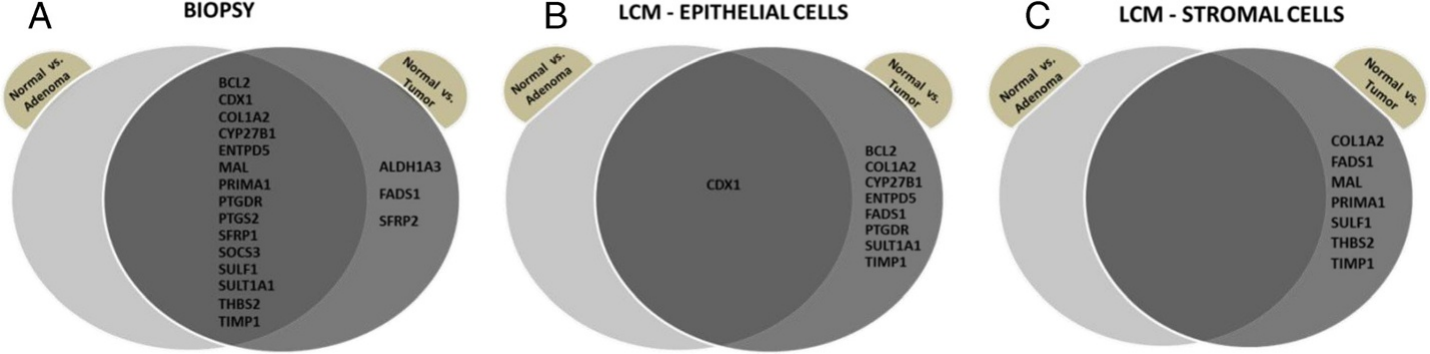
Summary of genes with altered expression levels in the analysed samples. Venn diagrams display genes that exhibit significantly altered gene expression patterns (*p* < 0.05) in (**a**) colon biopsy samples, (**b**) laser capture microdissected (LCM) epithelial cells, and (**c**) stromal cells in the normal versus adenoma, normal versus tumour comparisons and their intersections. The majority of gene expression changes could be detected in biopsy samples, while LCM epithelial and stromal cells show fewer altered transcript levels, primarily in normal vs. tumour comparison

Genes potentially regulated by DNA methylation were selected on the basis of whole genome gene expression data from previously performed microarray experiments of 49 normal, 49 adenoma, and 49 tumour biopsy samples [9]. Based on Kendall analysis, a set of 18 transcripts was selected showing continuously altering expression (*p* ≤ 0.01) in the biopsy samples along the adenoma-carcinoma sequence (Table 1). Along colorectal adenoma-carcinoma progression, the following genes showed downregulation: *BCL2, CDX1, ENTPD5, MAL, PRIMA1, PTGDR, SFRP1*, and *SULT1A1* while the following genes showed upregulation: *ALDH1A3, COL1A2, CYP27B1, FADS1, PTGS2, SFRP2, SOCS3, SULF1, THBS2*, and *TIMP1*. Gene expression alteration of *BCL2, CDX1, CYP27B1, ENTPD5, MAL, PRIMA1, PTGDR, PTGS2, SFRP1, SOCS3 SULT1A1,* and *TIMP1* were found to be significant (*p* < 0.05) in the adenoma versus healthy and also in the tumour versus healthy comparison. In addition, *ALDH1A3, COL1A2, FADS1, SFRP2, SULF1,* and *THBS2* were found to be significantly (*p* < 0.01) differentially expressed in tumour samples but not in adenomas compared to healthy samples (Fig. 1, Table 2, Additional file 3: Figure S1, Additional file 4: Table S3).

**Table 2:** Gene expression data of biopsies and laser microdissected (LCM) colon epithelial cells

	Normal	Adenoma	CRC	*P*-value (N vs. Ad)	*P*-value (N vs. CRC)	*P*-value (Ad vs. CRC)
	Mean normalised intensity values ± SD	Mean normalised intensity values ± SD	Mean normalised intensity values ± SD			
Biopsy samples						
203180_at (ALDH1A3)	6.36 ± 0.92	6.53 ± 0.86	7.29 ± 1.45	0.34	*p* < 0.01	*p* < 0.01
203685_at (BCL2)	8.90 ± 0.52	7.96 ± 1.08	6.98 ± 1.22	*p* < 0.01	*p* < 0.01	*p* < 0.01
206430_at (CDX1)	11.70 ± 0.28	11.17 ± 0.35	10.84 ± 0.73	*p* < 0.01	*p* < 0.01	*p* < 0.01
202404_s_at (COL1A2)	9.75 ± 0.78	10.25 ± 1.11	12.31 ± 1.67	0.01	*p* < 0.01	*p* < 0.01
205676_at (CYP27B1)	2.55 ± 0.10	2.88 ± 0.54	3.15 ± 0.95	*p* < 0.01	*p* < 0.01	0.09
1554094_at (ENTPD5)	6.41 ± 0.70	4.56 ± 0.79	4.15 ± 1.29	*p* < 0.01	*p* < 0.01	0.06
208963_x_at (FADS1)	4.82 ± 0.70	5.05 ± 1.14	6.36 ± 1.65	0.22	*p* < 0.01	*p* < 0.01
204777_s_at (MAL)	6.02 ± 0.62	4.86 ± 0.37	4.88 ± 0.59	*p* < 0.01	*p* < 0.01	0.86
230087_at (PRIMA1)	4.09 ± 0.97	2.20 ± 0.61	2.31 ± 0.83	*p* < 0.01	*p* < 0.01	0.47
234165_at (PTGDR)	5.84 ± 1.08	3.91 ± 1.75	2.81 ± 0.85	*p* < 0.01	*p* < 0.01	*p* < 0.01
204748_at (PTGS2)	4.96 ± 1.33	6.84 ± 2.50	9.88 ± 2.74	*p* < 0.01	*p* < 0.01	*p* < 0.01
202036_s_at (SFRP1)	4.05 ± 1.47	2.18 ± 0.62	2.51 ± 1.28	*p* < 0.01	*p* < 0.01	0.11
223121_s_at (SFRP2)	2.87 ± 0.11	2.92 ± 0.28	3.94 ± 1.74	0.29	*p* < 0.01	*p* < 0.01
227697_at (SOCS3)	6.10 ± 1.11	7.42 ± 1.65	9.81 ± 2.06	*p* < 0.01	*p* < 0.01	*p* < 0.01
212353_at (SULF1)	6.29 ± 1.04	7.12 ± 1.16	9.23 ± 2.15	*p* < 0.01	*p* < 0.01	*p* < 0.01
215299_x_at (SULT1A1)	11.94 ± 0.53	10.40 ± 0.85	10.34 ± 0.95	*p* < 0.01	*p* < 0.01	0.72
203083_at (THBS2)	2.61 ± 0.44	3.46 ± 1.54	7.67 ± 3.13	*p* < 0.01	*p* < 0.01	*p* < 0.01
201666_at (TIMP1)	10.29 ± 0.75	12.20 ± 0.69	12.80 ± 1.04	*p* < 0.01	*p* < 0.01	*p* < 0.01
LCM - colon epithelial cells						
203180_at (ALDH1A3)	3.44 ± 0.83	3.35 ± 0.34	3.61 ± 0.73	0.80	0.72	0.45
203685_at (BCL2)	7.02 ± 0.64	6.94 ± 1.56	4.52 ± 2.00	0.91	*p* < 0.05	*p* < 0.05
206430_at (CDX1)	10.35 ± 0.57	9.51 ± 0.47	9.38 ± 0.53	*p* < 0.05	*p* < 0.05	0.65
202404_s_at (COL1A2)	4.27 ± 1.50	3.28 ± 0.69	7.55 ± 1.02	0.18	*p* < 0.01	*p* < 0.01
205676_at (CYP27B1)	2.75 ± 0.04	2.79 ± 0.05	3.07 ± 0.24	0.10	*p* < 0.01	*p* < 0.05
1554094_at (ENTPD5)	4.51 ± 0.68	3.80 ± 0.69	2.51 ± 0.08	0.10	*p* < 0.01	*p* < 0.01
208963_x_at (FADS1)	3.24 ± 0.21	3.18 ± 0.17	4.44 ± 1.49	0.57	0.08	0.07
204777_s_at (MAL)	2.38 ± 0.17	2.30 ± 0.00	2.31 ± 0.02	0.29	0.34	0.30
230087_at (PRIMA1)	2.63 ± 0.29	3.88 ± 1.08	2.43 ± 0.12	*p* < 0.05	0.14	*p* < 0.01
234165_at (PTGDR)	4.71 ± 0.73	3.77 ± 1.02	2.39 ± 0.09	0.10	*p* < 0.01	*p* < 0.01
204748_at (PTGS2)	2.61 ± 0.15	2.68 ± 0.24	2.51 ± 0.04	0.59	0.14	0.13
202036_s_at (SFRP1)	2.71 ± 0.35	2.76 ± 0.33	2.54 ± 0.00	0.80	0.27	0.13
223121_s_at (SFRP2)	2.35 ± 0.09	2.36 ± 0.16	2.30 ± 0.00	0.81	0.28	0.34
227697_at (SOCS3)	4.97 ± 1.91	2.82 ± 0.43	4.33 ± 1.69	*p* < 0.05	0.55	0.06
212353_at (SULF1)	3.08 ± 0.81	2.73 ± 0.09	3.99 ± 1.44	0.32	0.21	0.06
215299_x_at (SULT1A1)	9.35 ± 0.31	8.57 ± 1.16	7.20 ± 0.95	0.14	*p* < 0.01	*p* < 0.05
203083_at (THBS2)	2.58 ± 0.14	2.53 ± 0.00	3.54 ± 1.43	0.48	0.13	0.12
201666_at (TIMP1)	4.11 ± 1.06	6.54 ± 1.62	7.85 ± 0.57	*p* < 0.05	*p* < 0.01	0.09

In order to investigate the cellular origin of altered gene expression of the analysed transcript set during colorectal cancer formation, laser capture microdissection was applied to separate epithelial and stromal cells from the colonic mucosa. Significantly altered expression (*p* < 0.05) of *SOCS3* and *PRIMA1* could be detected in epithelial cells from normal versus adenomatous samples. Gene expression changes of *BCL2, CYP27B1, COL1A2, FADS1,* and *SULT1A1* were significant (*p* < 0.05) only in tumours compared to healthy samples, while *CDX1, ENTPD5, PTGDR ,*and *TIMP1* showed gene expression difference in both normal vs. adenoma and normal vs. tumour comparisons (Fig. 1, Table 2, Additional file 4: Table S3).

No significant gene expression alterations could be detected in the stromal cells isolated from adenomas compared to the normals, but COL1A2, FADS1, MAL, PRIMA1, SULF1, THBS2, TIMP1 genes’ transcripts showed significant differences (*p* < 0.05) in logFc values for the tumour versus normal comparison (Fig. 1; Additional file 4: Table S3). As stromal cells showed the fewest gene expression alterations, we further focused on biopsy and laser microdissected epithelial samples.

### Demethylation treatment on colon adenocarcinoma cell lines

**Fig. 2:**
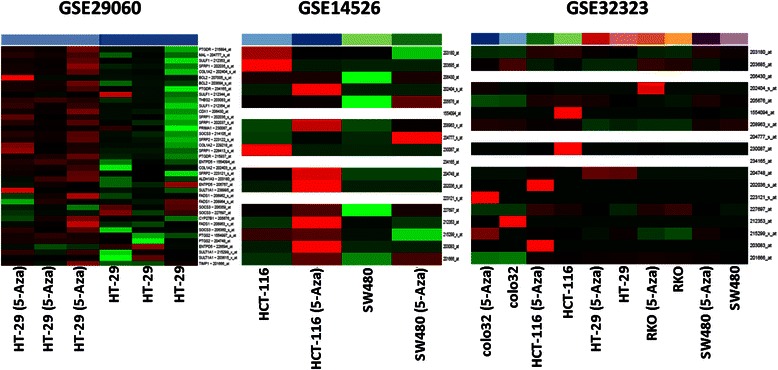
Heat map of gene expression data of the selected marker set in 5-aza-2’-deoxycytidine-treated human colon adenocarcinoma cells (GSE29060; GSE14526; GSE32323). Intensity values on the colour scale were as follows: red – high intensity, black – intermediate intensity, green – low intensity. Demethylation treatment resulted in varying degrees of upregulation of certain transcripts

Gene expression of the selected marker set was analysed on data sets containing control and 5-Aza treated colon adenocarcinoma cell lines. According to GSE29060 data, in HT-29 adenocarcinoma cells after a demethylation treatment 4 transcripts showed a minimally decreased expression *(TIMP1, FADS1, CYP27B* and *SULT1A1*), while *PTGS2* was found to be upregulated. HCT-116 cells showed higher re-expression of the selected genes, as *PTGS2, THBS2* and *TIMP1* also showed upregulation (1 < logFc_control-treated_) and *TIMP1* was also upregulated in 5-Aza treated SW480 cells according to GSE14526. Among the 5 CRC cell lines of GSE32323 *SULT1A1* in Colo32 cells, *PTGS2* in HCT-116 cells, *ALDH1A3* and *SOCS3* in HT-29 cells and *ALDH1A3* and *TIMP1* in SW480 cells showed remarkable upregulation after demethylation treatment (Fig. 2, Additional file 4: Table S3).

### DNA methylation analysis

DNA methylation was assessed in human colonic samples using two different pyrosequencing systems. Firstly, routinely collected biopsy samples and macrodissected specimens naturally containing both epithelial and stromal cells were analysed. Among the 18 analysed markers (Table 1), DNA methylation was significantly (*p* < 0.05) altered for six loci belonging to four genes, in which *COL1A2, SFRP2, SOCS3* showed hypermethylation and *THBS2* showed hypomethylation both in AD and in CRC samples compared to NAT. Three additional genes, *BCL2, PRIMA1, and PTGDR* showed hypermethylation only in tumour samples (Table 3, Additional file 5: Figure S2).

**Table 3:** DNA methylation data of biopsies, macrodissected samples and laser microdissected (LCM) colon epithelial cells

	Normal	Adenoma	CRC	*P*-value (N vs. Ad)	*P*-value (N vs. CRC)	*P*-value (Ad vs. CRC)
	Mean DNA methylation % ± SD	Mean DNA methylation % ± SD	Mean DNA methylation % ± SD			
Biopsy samples						
ALDH1A3_assay 1	4.33 ± 2.23	3.59 ± 1.92	5.74 ± 6.26	0.31	0.36	0.21
BCL2_assay 1	3.22 ± 1.12	3.50 ± 2.21	9.74 ± 13.41	0.63	*p* < 0.05	0.09
BCL2_assay 2	0.91 ± 0.72	0.42 ± 0.41	0.73 ± 0.59	*p* < 0.05	0.44	0.11
CDX1_assay 1	21.96 ± 9.38	19.41 ± 8.76	16.01 ± 9.83	0.42	0.08	0.32
COL1A2 assay 2	22.67 ± 5.62	37.37 ± 14.36	39.52 ± 18.02	*p* < 0.01	*p* < 0.01	0.72
COL1A2_assay 1	8.65 ± 3.63	9.77 ± 6.13	15.31 ± 12.57	0.51	*p* < 0.05	0.14
CYP27B1_assay 1	10.71 ± 3.14	8.53 ± 4.19	11.37 ± 6.76	0.09	0.71	0.18
CYP27B1_assay 2	4.03 ± 4.16	2.71 ± 1.69	6.45 ± 10.56	0.26	0.36	0.19
CYP27B1_assay 4	45.12 ± 2.66	44.50 ± 2.08	46.70 ± 8.04	0.46	0.42	0.31
ENTPD5_assay 1	2.17 ± 0.62	1.78 ± 0.91	2.93 ± 3.32	0.15	0.35	0.20
FADS1_assay 1	0.75 ± 0.44	0.46 ± 0.23	6.98 ± 10.82	*p* < 0.05	*p* < 0.05	*p* < 0.05
MAL_assay 1	16.46 ± 16.12	38.87 ± 26.52	50.42 ± 26.99	*p* < 0.01	*p* < 0.01	0.25
PRIMA1_assay 1	7.25 ± 3.15	9.88 ± 10.60	28.91 ± 22.65	0.30	*p* < 0.01	*p* < 0.01
PRIMA1_assay 2	3.99 ± 1.32	6.49 ± 5.60	22.29 ± 20.14	0.06	*p* < 0.01	*p* < 0.01
PRIMA1_assay 3	37.96 ± 13.57	48.13 ± 20.69	56.43 ± 21.17	0.11	*p* < 0.01	0.30
PRIMA1_assay 4	8.30 ± 7.95	17.78 ± 13.26	26.82 ± 25.00	*p* < 0.05	*p* < 0.01	0.23
PTGDR assay 1	5.79 ± 1.84	4.09 ± 1.85	11.12 ± 9.36	*p* < 0.05	*p* < 0.05	*p* < 0.01
PTGDR assay 2	11.71 ± 3.51	8.01 ± 4.50	15.58 ± 6.48	*p* < 0.05	*p* < 0.05	*p* < 0.01
PTGDR_assay 3	6.47 ± 9.27	3.61 ± 4.63	10.47 ± 9.51	0.28	0.22	*p* < 0.05
PTGS2_assay 1	8.82 ± 3.47	12.15 ± 15.01	10.80 ± 13.16	0.34	0.52	0.80
PTGS2_assay 2	4.33 ± 9.01	5.83 ± 10.49	5.64 ± 8.48	0.65	0.67	0.96
SFRP1_assay 1	39.60 ± 18.46	60.82 ± 20.41	54.49 ± 18.47	*p* < 0.01	*p* < 0.05	0.38
SFRP2_assay 1	14.84 ± 4.38	38.52 ± 20.06	44.44 ± 20.05	*p* < 0.01	*p* < 0.01	0.43
SFRP2_assay 2	20.14 ± 4.48	39.33 ± 17.77	48.04 ± 16.20	*p* < 0.01	*p* < 0.01	0.17
SOCS3_assay 1	4.96 ± 1.52	7.57 ± 6.18	12.16 ± 12.01	0.08	*p* < 0.05	0.20
SOCS3_assay 2	18.24 ± 7.68	49.27 ± 20.49	49.43 ± 18.81	*p* < 0.01	*p* < 0.01	0.98
SOCS3_assay 3	8.82 ± 16.20	21.75 ± 21.72	25.79 ± 22.22	0.05	*p* < 0.05	0.62
SULF1_assay 1	5.32 ± 2.16	6.80 ± 11.30	9.60 ± 8.91	0.57	*p* < 0.05	0.46
SULF1_assay 2	4.60 ± 4.02	7.98 ± 10.78	11.28 ± 14.82	0.21	0.06	0.49
SULT1A1_assay 1	5.51 ± 4.35	3.25 ± 1.99	7.58 ± 9.46	0.07	0.39	0.09
SULT1A1_assay 2	42.68 ± 5.54	44.86 ± 9.49	48.80 ± 10.07	0.40	*p* < 0.05	0.28
SULT1A1_assay 3	3.11 ± 2.94	2.14 ± 1.66	6.34 ± 6.55	0.26	0.06	*p* < 0.05
THBS2_assay 1	18.17 ± 5.03	26.47 ± 13.79	28.36 ± 16.92	*p* < 0.05	*p* < 0.05	0.74
THBS2_assay 2	91.32 ± 2.37	85.64 ± 6.49	83.73 ± 6.83	*p* < 0.01	*p* < 0.01	0.44
THBS2_assay 3	89.68 ± 1.80	87.51 ± 3.55	83.02 ± 6.69	*p* < 0.05	*p* < 0.01	*p* < 0.05
THBS2_assay 4	22.32 ± 10.74	27.97 ± 13.58	25.47 ± 15.20	0.18	0.48	0.64
TIMP1_assay 1	19.54 ± 14.39	16.65 ± 14.45	13.96 ± 10.07	0.56	0.21	0.56
LCM – colon epithelial cells						
ALDH1A3_assay 1	8.30 ± 3.96	4.02 ± 2.56	3.60 ± 3.49	0.08	0.08	0.83
BCL2_assay 1	3.14 ± 1.18	3.30 ± 1.19	4.26 ± 2.58	0.84	0.40	0.47
BCL2_assay 2	4.53 ± 7.59	0.32 ± 0.35	0.33 ± 0.47	0.24	0.30	0.99
CDX1_assay 1	11.26 ± 19.16	1.64 ± 1.16	2.43 ± 2.43	0.29	0.40	0.54
COL1A2 assay 2	19.18 ± 6.95	61.72 ± 18.25	53.46 ± 18.52	*p* < 0.01	*p* < 0.01	0.50
COL1A2_assay 1	6.46 ± 3.34	14.06 ± 6.37	22.84 ± 7.00	*p* < 0.05	*p* < 0.01	0.07
CYP27B1_assay 1	10.20 ± 12.19	3.28 ± 0.94	7.88 ± 7.06	0.24	0.75	0.19
CYP27B1_assay 2	7.14 ± 8.88	0.50 ± 0.31	6.38 ± 8.64	0.13	0.90	0.17
CYP27B1_assay 4	43.56 ± 2.13	44.90 ± 3.89	45.90 ± 5.26	0.52	0.38	0.74
ENTPD5_assay 1	1.68 ± 0.56	1.74 ± 0.11	1.88 ± 0.60	0.80	0.62	0.62
FADS1_assay 1	12.76 ± 17.16	4.70 ± 7.95	2.94 ± 5.80	0.37	0.26	0.70
MAL_assay 1	11.76 ± 10.80	61.16 ± 40.61	84.68 ± 11.92	*p* < 0.05	*p* < 0.01	0.25
PRIMA1_assay 1	4.00 ± 0.85	32.56 ± 31.71	26.42 ± 33.03	0.08	0.17	0.77
PRIMA1_assay 2	2.34 ± 1.13	29.82 ± 22.74	29.45 ± 35.82	*p* < 0.05	0.13	0.99
PRIMA1_assay 3	21.70 ± 25.92	73.64 ± 28.56	93.55 ± 3.06	*p* < 0.05	*p* < 0.01	0.21
PRIMA1_assay 4	0.62 ± 0.73	43.61 ± 29.53	59.91 ± 31.31	*p* < 0.05	*p* < 0.01	0.42
PTGDR assay 1	2.95 ± 0.80	3.16 ± 2.52	28.94 ± 10.72	0.87	*p* < 0.01	*p* < 0.01
PTGDR assay 2	6.40 ± 3.82	3.56 ± 1.26	23.81 ± 10.40	0.15	*p* < 0.01	*p* < 0.01
PTGDR_assay 3	1.63 ± 3.23	1.14 ± 1.45	25.72 ± 20.81	0.77	*p* < 0.05	*p* < 0.05
PTGS2_assay 1	5.80 ± 6.58	2.33 ± 0.33	3.05 ± 1.00	0.27	0.44	0.17
PTGS2_assay 2	9.66 ± 8.80	0.83 ± 0.80	18.19 ± 36.43	0.06	0.62	0.32
SFRP1_assay 1	19.03 ± 25.20	78.72 ± 23.36	91.08 ± 5.91	*p* < 0.01	*p* < 0.01	0.28
SFRP2_assay 1	12.53 ± 5.01	55.95 ± 31.32	90.78 ± 1.56	*p* < 0.05	*p* < 0.01	0.06
SFRP2_assay 2	16.34 ± 9.82	68.90 ± 27.80	85.16 ± 2.56	*p* < 0.01	*p* < 0.01	0.29
SOCS3_assay 1	3.86 ± 0.84	6.57 ± 5.39	25.07 ± 14.20	0.30	*p* < 0.05	*p* < 0.05
SOCS3_assay 2	14.28 ± 8.30	68.61 ± 35.98	90.36 ± 8.66	*p* < 0.05	*p* < 0.01	0.28
SOCS3_assay 3	2.59 ± 2.72	55.24 ± 43.52	85.04 ± 12.02	*p* < 0.05	*p* < 0.01	0.23
SULF1_assay 1	4.62 ± 4.06	7.52 ± 8.25	16.12 ± 17.25	0.50	0.18	0.34
SULF1_assay 2	4.75 ± 4.58	8.35 ± 12.00	8.46 ± 4.78	0.59	0.31	0.99
SULT1A1_assay 1	2.54 ± 1.05	4.63 ± 3.81	4.02 ± 2.15	0.27	0.20	0.76
SULT1A1_assay 2	45.35 ± 7.51	38.07 ± 4.36	56.53 ± 26.33	0.10	0.39	0.16
SULT1A1_assay 3	13.90 ± 16.07	7.69 ± 8.11	7.87 ± 12.23	0.47	0.57	0.98
THBS2_assay 1	13.64 ± 6.67	45.55 ± 25.89	49.61 ± 8.64	*p* < 0.05	*p* < 0.01	0.75
THBS2_assay 2	90.83 ± 2.02	84.92 ± 8.28	83.33 ± 10.87	0.16	0.17	0.80
THBS2_assay 3	90.16 ± 2.37	87.54 ± 3.22	78.65 ± 8.85	0.18	*p* < 0.05	0.07
THBS2_assay 4	24.84 ± 18.37	42.88 ± 8.47	42.48 ± 18.50	0.11	0.33	0.97
TIMP1_assay 1	32.62 ± 21.75	15.35 ± 20.90	5.35 ± 2.27	0.24	*p* < 0.05	0.32

Interestingly, two of the analysed regions in the *THBS2* promoter conferred hypomethylation along tumour formation, while the third locus examined showed significant hypermethylation in tumours compared to NAT.

### Unsupervised clustering of genes with DNA hypermethylation

**Fig. 3:**
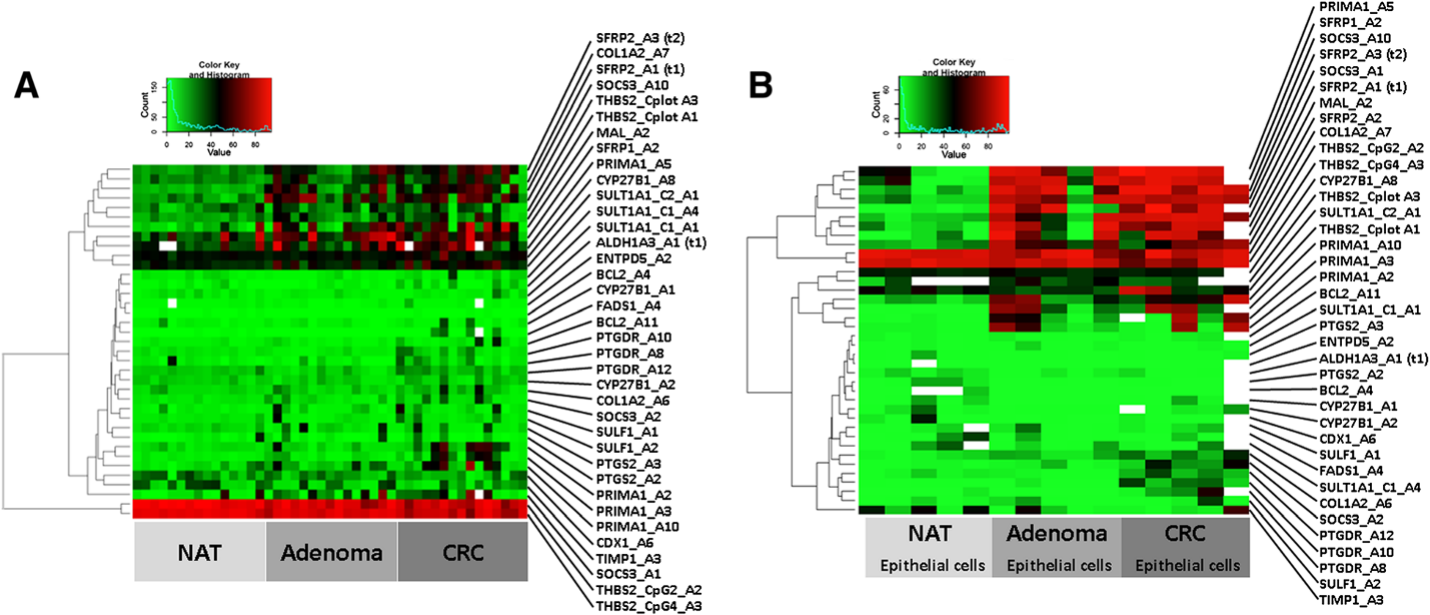
Heatmap representing level of DNA methylation in **a**) NAT, AD, and CRC biopsies and macrodissected samples and in **b**) NAT, AD and CRC LCM epithelial cells. Intensity values on the colour scale were as follows: red - high intensity, black - intermediate intensity, green - low intensity. Samples are shown in columns, selected genes are in rows. Similar DNA methylation pattern could be found in both sample types, as *PRIMA1*, *SFRP1*, *SFRP2*, *MAL*, *SOCS3*, *CYP27B1*, *COL1A2* and *SULT1A1* showed relatively high DNA methylation levels in colon biopsies and LCM epithelial cells

Unsupervised hierarchical clustering of DNA methylation data revealed three groups of markers in biopsy and macrodissected sample groups. The first group of genes (*SFRP2, COL1A2, THBS2, SOCS3, CYP27B1, SULT1A1, PRIMA1* and *MAL*) showed a relatively high degree of DNA methylation already in AD and also in CRC samples. The second group included most markers and did not show remarkable difference among different sample groups, while the third minor cluster included only two *THBS2* loci with high methylation levels across all samples (Fig. 3a). Unsupervised hierarchical clustering of LCM epithelial cells revealed similar relationships to those in biopsy and macrodissected samples above. Certain genes showed relatively high DNA methylation levels in both biopsies and epithelial cells in adenoma and cancer cases, as *PRIMA1, SFRP1, SFRP2, MAL, SOCS3, CYP27B1, COL1A2* and *SULT1A1. THBS2* showed high methylation levels across all samples. The second major marker group included most genes and did not show remarkable difference between the different sample groups (Fig. 3b).

### miRNA analysis

**Fig. 4:**
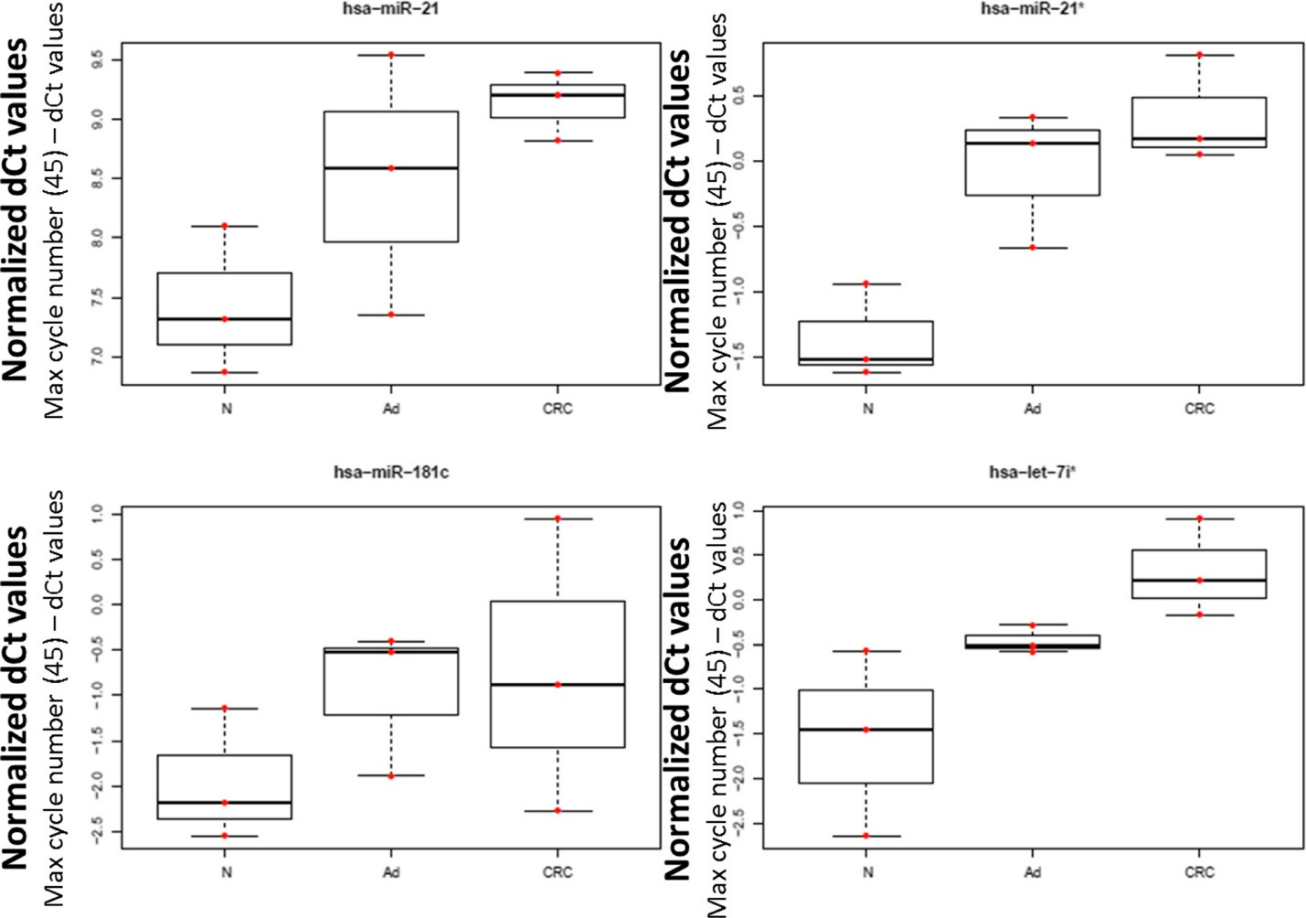
Normalised Ct values of selected miRNAs (hsa-miR-21, hsa-miR-21*, hsa-miR-181c, hsa-let-7i*) targeting the selected marker set. Raw Ct data were substracted from the maximal qPCR cycle number (45) and data were normalised with interplate calibrators and also with miR-423-5p Ct values. Red dots represent individual miRNA normalised Ct values, box plots represent median and standard deviation of the data

We used the miRWALK database to predict miRNAs that could target genes of our selected set. Multiple miRNAs could be predicted using the miRWALK ’Validated Target’ *in silico* searching application. Certain groups of miRNAs were predicted to target more genes analysed in our present study; miR-21 (predicted for *BCL2, MAL, PTGS2, SFRP1, SOCS3*) expression was found to be remarkably upregulated in CRC compared to NAT (Fig. 4). Furthermore, miR-21* (predicted for *BCL2, MAL, SFRP1, SOCS3, PTGS2*), miR-181c (predicted for *ALDH1A3, BCL2, MAL*), and let-7i* (predicted for *BCL2, CYP27B1,* and *SOCS3*) were also found to be upregulated in AD and CRC samples (Fig. 4).

### Immunohistochemistry

**Fig. 5:**
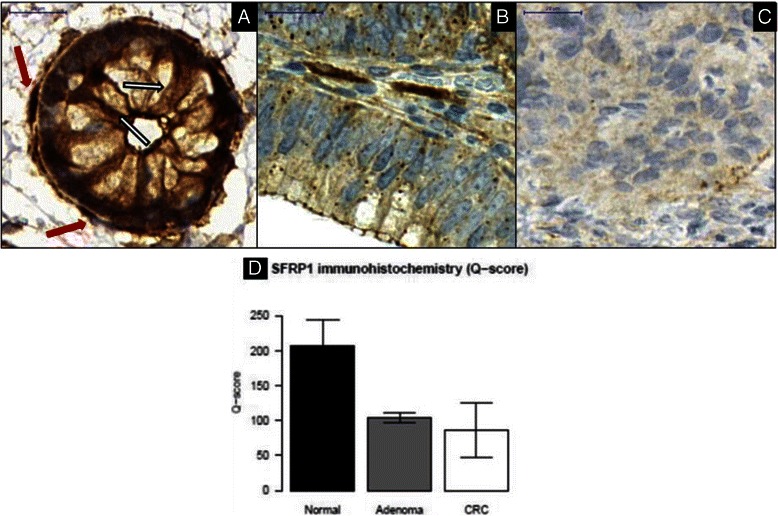
Continuously decreasing SFRP1 protein expression could be observed along colorectal adenoma-carcinoma development in epithelial/CRC compartment of NAT (**a**), AD (**b**), and CRC (**c**) samples. SFRP1 protein expression of healthy epithelial cells (**a**, *white arrows*) was compared to that in endogenous myofibroblasts (**a**, *red arrows*) with strong (+3) immunopositivity (digital microscopy images, 90x magnification, scale bar: 20 μm). Semiquantitative immunohistochemistry results (Q-score values) of NAT, AD, and CRC specimens are summarised as bar charts with whiskers representing standard deviation (**d**)

Colonic FFPE tissue samples were immunostained for SFRP1. In NAT epithelium, moderate diffuse cytoplasmic staining (+2) could be detected (Fig. 5a, white arrows) in contrast to adjacent myofibroblasts (we identified they by their localisation and morphology) with strong diffuse immunostaining (+3) (Fig. 5a, red arrows). In tubular AD samples, weak diffuse cytoplasmic protein expression (+1) was accompanied by strong and spotted immunostaining (+2/+3) (Fig. 5b). The majority of CRC cases (9 out of 10 cases) showed weak (+1) or no (0) SFRP1 immunostaining (Fig. 5c). According to Q-score values used for semiquantitative immunohistochemistry analysis, the overall SFRP1 protein expression decreased along the colorectal adenoma-carcinoma sequence (Fig. 5).

## Discussion

The goal of this study was to identify DNA methylation and miRNA markers associated with the sequence of adenoma-carcinoma formation leading to CRC. The candidate markers were selected based on whole genome gene expression array data, DNA methylation analysis, and *in silico* prediction and validation of miRNA expression.

The study identified set of 18 transcripts showing continuous gene expression alterations that correlated with CRC progression. Microarray experiments revealed 12 genes (*BCL2, CDX1, CYP27B1, ENTPD5, MAL, PRIMA1, PTGDR, PTGS2, SFRP1, SOCS3, SULT1A1,* and *TIMP1*) with significantly different transcriptional activities in AD compared to NAT controls, while 6 genes (*ALDH1A3, COL1A2, FADS1, SFRP1, SULF1,* and *THBS2*) showed unique gene expression alterations only in CRC samples. More specifically, looking at cellular components of the abovementioned stages of CRC formation, the results showed that epithelial cells in AD express decreased amounts of *SOCS3* and *PRIMA1,* whereas those in CRC express less *BCL2, CYP27B1, COL1A2, FADS1,* and *SULT1A1.*

Demethylation treatment of colon adenocarcinoma cell lines led to varying degrees of upregulation of certain transcripts. In HT-29 cell line *ALDH1A3* and *SOCS3* was found to be upregulated by 0.5 μM 5-Aza. Interestingly, in HCT-116 cells *PTGS2;* and in SW480 cell line *TIMP1* showed higher expression after 0.5 and 3 μM 5-Aza treatments, as well.

From the resulting marker set, *COL1A2*, *SFRP2*, and *SOCS3* were hypermethylated and *THBS2* was hypomethylated in both AD and CRC samples compared to NAT. Based on the literature, hypermethylation of *COL1A2* was confirmed in head and neck cancer [20], melanoma [21], and bladder cancer [22]. This is suggestive that *COL1A2* may contribute to the formation of various cancers by modulating cell proliferation and migration. In the gastrointestinal tract, expression of *COL1A2* may be associated with endothelial-to-mesenchymal transition [23]. Collagen production of carcinoma cells decreases during oncogenic transformation [24]; and, hypermethylation of *COL1A2* was confirmed in several CRC cell lines (HCT 116, SW480, and SW620) as well as in primary CRC tissues [25]. *SFRP2* is a member of the well-known inhibitors of Wnt pathway, abnormal activation of which (e.g. via *APC* mutation or beta-catenin translocation) is a frequent and early event in the genesis of CRC [26]. It has already been shown to be hypermethylated in colorectal cancer cell lines (e.g. HCT116) as well as primary CRC [27, 28]. Furthermore, it has recently been recognised as a promising and sensitive marker of stool-based screening of CRC [26]. *SOCS3* is a negative regulator of the JAK-STAT3 pathway; therefore, it may effect cell proliferation and cell cycle [29]. Mutational analysis of the gene revealed no marked association between *SOCS3* promoter region polymorphisms and the risk of developing metastatic colorectal cancer [30]. Epigenetic inactivation of *SOCS3* was reported in human malignant melanomas and glioblastoma multiforme [31, 32]. Reduced gene expression of *SOCS3* was found in the colitis ulcerosa (UC) to CRC progression from low-grade dysplasia to CRC. Related to this, DNA methylation of *SOCS3* could also be detected in colonic biopsies of UC-CRC patients but not from healthy controls or from inactive UC patients [33, 34]. *THBS2* hypermethylation might be responsible for altered expression of thrombospondin-2 protein in ovarian cancer and endometrial adenocarcinomas [35]. Thrombospondin-2 is an antiangiogenetic factor in CRC and its expression was associated with angiogenesis and metastasis formation inhibition in CRC [36].

The set of *BCL2, PRIMA1,* and *PTGDR* showed hypermethylation only in CRC. *BCL2* (B-cell CLL/lymphoma 2) is an apoptotic inhibitor. Its hypermethylation was documented in breast cancer [37] and bladder cancer [38]. Bcl-2 protein plays a role in CRC formation [39] and has a reduced expression in CRCs with microsatellite instability [40]. DNA hypermethylation of *BCL2* was detected in CRC cases; however, there was no relationship between gene expression and methylation of specific CpG sites [41]. *PRIMA1* encodes a membrane protein anchoring acetylcholinesterase to cell membranes [42]. Its promoter hypermethylation was detected in major depressive disorder with a concomitant decrease in gene expression [43]. It has not yet been associated with CRC development. Decreased mRNA expression levels of *PTGDR* genes in colorectal AD and CRC caused by DNA methylation were previously described [8].

In summary *MAL, PRIMA1, PTGDR* and *SFRP1* showed a downregulation of gene expression and in parallel increasing DNA methylation level that correlated with CRC development. Meanwhile, *BCL2*, *CDX1*, *ENTPD5* and *SULT1A1* dowregulation was not accompanied with significant DNA methylation changes; thus, other regulatory processes should be further investigated to understand these changes in gene expression.

After DNA methylation analysis of candidate genes with altered gene expression, the potential influence of DNA methylation on the protein level was also investigated. Significantly decreasing protein levels of *SFRP1* could be observed along the adenoma-carcinoma sequence. This result is in accordance with the literature, as epigenetic regulation of *SFRP1* can lead to decreased protein levels [44, 45].

On a limited sample set miRNAs with upregulation along the AD-CRC sequence were also identified. miR-21 was found to be remarkably upregulated in AD and CRC samples compared to NAT controls. On the basis of *in silico* prediction miR-21 can target genes showing no remarkable alteration in their promoter methylation (e.g. *BCL2, MAL, PTGS2*) during CRC development, that might influence their gene expression levels. miR-21 is known to play role in tumour formation and was also found to be upregulated in CRC tissues along tumour formation [46, 47]. The expression level of miR-21 is elevated both in colorectal adenomas and cancers, and the degree of upregulation correlates with more advanced stages of CRC [7]. This small non-coding RNA could have a fundamental role in the progression of CRC, as elevated level of miR-21 was found to be predictive of poor survival [48], that may increase proliferation, migration and invasion. In CRC cell lines with the EMT phenotype the expression of miR-21 oncomiR is regulated by AP-1 and ETS transciption factors and also by epigenetic factors. Activating histone modifications (H3K3me3, H3K914ac, H3K27ac), but no inactivating were detected on miR-21 promoter region [49]. These epigenetic mechanisms can affect the binding affinity of transcription factors to the miR-21 promoter regulating its expression level. Upregulated miR-181 in CRC cases might also influence gene expression level of the Bcl-2 family members [50].

## Conclusion

In summary, we identified 18 transcripts with changes in gene expression that correlate with CRC development. On the basis of genome-wide gene expression-based screening we could identify genes potentially downregulated by promoter hypermethylation. Silencing of the markers identified in our study by hypermethylation or miRNA upregulation can result in reduced gene expression leading to decreased protein levels contributing to CRC formation.

## Additional files

Additional file 1: Table S1. Clinical data of the analysed gene expression datasets (GSE4183, GSE37364, GSE10714 and GSE15960). (DOC 249 kb)
Additional file 2: Table S2. Clinical data of patients involved in the study. (DOCX 24 kb)
Additional file 3: Figure S1. Gene expression of the selected marker set in normal (*n* = 49), adenoma (*n* = 49), and tumour (*n* = 49) biopsy samples. Results are represented as pairwise box plots showing single gene expession in the biopsy and in the laser microdissected epithelial cells from normal (*n* = 6), adenoma (*n* = 6), and tumour (*n* = 6). Red dots are normalised gene expression values, box plots represent median and standard deviation of the data. Asterisk (*) represents significance (*p* < 0.05) in adenoma and tumour samples compared to normals and double asterisk (**) represents significance (*p* < 0.05) in tumour samples compared to adenomas. Each box plot has individual scale of gene expression. Changes in the direction of gene expression was found to be similar in colon biopsies and in LCM epithelial cells. (PDF 296 kb)
Additional file 4: Table S3. Summary of gene expression and DNA methylation data. (XLS 49 kb)
Additional file 5: Figure S2. Box plot representation of DNA methylation alterations of analysed genes in biopsies and macrodissected specimens of NAT (*n* = 10), AD (*n* = 10), and CRC (*n* = 10) and in LCM epithelial cells of NAT (*n* = 5), AD (*n* = 5) and CRC (*n* = 5). Red dots are individual DNA methylation percent values, box plots represent median and standard deviation of the data. Asterisk (*) represents significance (*p* < 0.05) in adenoma and tumour samples compared to normals and double asterisk (**) represents significance (*p* < 0.05) in tumour samples compared to adenomas. Each box plot has individual scale of DNA methylation percent. Certain genes showe hypermethylation in colon biopsies and also in LCM epithelial cells (e.g. *MAL, PRIMA1, PTGDR, SFRP2*), while there were genes with no remarkable DNA methylation level alterations (e.g. *BCL2, CDX1, PTGS2, SULF1*). Macro = macrodissected samples, LCM = laser microdissected epithelial cells. (PDF 359 kb)

## Abbreviations

NAT: Normal tissue adjacent to tumour
AD: Adenoma
CRC: Colorectal cancer
LCM: Laser capture microdissection
CpG: CG dinucleotide
bcDNA: bisulfite-converted DNA
BS-PCR: Bisulfite-sequencing PCR
emPCR: emulsion-PCR
FFPE: Formalin-fixed and paraffin-embedded
TMA: Tissue microarray
