# Supplementary material for: DNA hypermethylation and decreased mRNA expression of MAL, PRIMA1, PTGDR and SFRP1 in colorectal adenoma and cancer
Source: BMC Cancer. 2015 Oct 19;15:736. doi: 10.1186/s12885-015-1687-x (PMC4612409; doi:10.1186/s12885-015-1687-x)
Supplement: Additional file 1: Table S1. — Clinical data of the analysed gene expression datasets (GSE4183, GSE37364, GSE10714 and GSE15960). (DOC 249 kb) [file 12885_2015_1687_MOESM1_ESM.doc]

**Supplementary Table 1.**

**GSE37364 –colorectal biopsy samples analyzed on HGU133Plus2.0 microarray**

| **Sample ID** | **Sample**  **type** | **Age range** | **Localization** | **Histology** | **TNM** | **Grade** | **Dukes** |
| --- | --- | --- | --- | --- | --- | --- | --- |
| **Normal** | | | | | | | |
| N1_GSE37364 | biopsy | 22-82 years | sigma | normal |  |  |  |
| N2_GSE37364 | biopsy |  | sigma | normal |  |  |  |
| N3_GSE37364 | biopsy |  | coecum | normal |  |  |  |
| N4_GSE37364 | biopsy |  | sigma | normal |  |  |  |
| N5_GSE37364 | biopsy |  | rectum | normal |  |  |  |
| N6_GSE37364 | biopsy |  | sigma | normal |  |  |  |
| N7_GSE37364 | biopsy |  | sigma | normal |  |  |  |
| N8_GSE37364 | biopsy |  | sigma | normal |  |  |  |
| N9_GSE37364 | biopsy |  | - | normal |  |  |  |
| N10_GSE37364 | biopsy |  | sigma | normal |  |  |  |
| N11_GSE37364 | biopsy |  | sigma | normal |  |  |  |
| N12_GSE37364 | biopsy |  | sigma | normal |  |  |  |
| N13_GSE37364 | biopsy |  | sigma | normal |  |  |  |
| N14_GSE37364 | biopsy |  | sigma, rectum | normal |  |  |  |
| N15_GSE37364 | biopsy |  | colon asc, sigma | normal |  |  |  |
| N16_GSE37364 | biopsy |  | colon asc, desc, sigma | normal |  |  |  |
| N17_GSE37364 | biopsy |  | colon asc, sigma | normal |  |  |  |
| N18_GSE37364 | biopsy |  | sigma | normal |  |  |  |
| N19_GSE37364 | biopsy |  | - | normal |  |  |  |
| N20_GSE37364 | biopsy |  | sigma, rectum | normal |  |  |  |
| N21_GSE37364 | biopsy |  | sigma, rectum | normal |  |  |  |
| N22_GSE37364 | biopsy |  | colon asc, rectum | normal |  |  |  |
| N23_GSE37364 | biopsy |  | sigma | normal |  |  |  |
| N24_GSE37364 | biopsy |  | sigma | normal |  |  |  |
| N25_GSE37364 | biopsy |  | sigma | normal |  |  |  |
| N26_GSE37364 | biopsy |  | - | normal |  |  |  |
| N27_GSE37364 | biopsy |  | sigma, rectum | normal |  |  |  |
| N28_GSE37364 | biopsy |  | - | normal |  |  |  |
| N29_GSE37364 | biopsy |  | - | normal |  |  |  |
| N30_GSE37364 | biopsy |  | colon desc, rectum | normal |  |  |  |
| N31_GSE37364 | biopsy |  | colon asc, transv, desc, sigma | normal |  |  |  |
| N32_GSE37364 | biopsy |  | colon asc, sigma | normal |  |  |  |
| N33_GSE37364 | biopsy |  | sigma, rectum | normal |  |  |  |
| N34_GSE37364 | biopsy |  | - | normal |  |  |  |
| N35_GSE37364 | biopsy |  | sigma, rectum | normal |  |  |  |
| N36_GSE37364 | biopsy |  | coecum, sigma | normal |  |  |  |
| N37_GSE37364 | biopsy |  | sigma | normal |  |  |  |
| N38_GSE37364 | biopsy |  | sigma | normal |  |  |  |
| **Low-grade dysplastic adenoma** | | | | | | | |
| LGD1_GSE37364 | biopsy | 50-88 years | sigma | tubulovillous adenoma |  |  |  |
| LGD2_GSE37364 | biopsy |  | coecum | tubulovillous adenoma |  |  |  |
| LGD3_GSE37364 | biopsy |  | colon desc | tubular adenoma |  |  |  |
| LGD4_GSE37364 | biopsy |  | sigma | villous adenoma |  |  |  |
| LGD5_GSE37364 | biopsy |  | colon transv | tubulovillous adenoma |  |  |  |
| LGD6_GSE37364 | biopsy |  | rectum | tubular adenoma |  |  |  |
| LGD7_GSE37364 | biopsy |  | transversum | tubular adenoma |  |  |  |
| LGD8_GSE37364 | biopsy |  | sigma | tubular adenoma |  |  |  |
| LGD9_GSE37364 | biopsy |  | sigma | tubular adenoma |  |  |  |
| LGD10_GSE37364 | biopsy |  | colon asc | tubular adenoma |  |  |  |
| LGD11_GSE37364 | biopsy |  | sigma | tubulovillous adenoma |  |  |  |
| LGD12_GSE37364 | biopsy |  | colon asc | tubular adenoma |  |  |  |
| LGD13_GSE37364 | biopsy |  | sigma | tubulovillous adenoma |  |  |  |
| LGD14_GSE37364 | biopsy |  | rectosigmoideum | tubular adenoma |  |  |  |
| LGD15_GSE37364 | biopsy |  | rectum | tubulovillous adenoma |  |  |  |
| LGD16_GSE37364 | biopsy |  | rectum | tubulovillous adenoma |  |  |  |
| **High-grade dysplastic adenoma** | | | | | | | |
| HGD1_GSE37364 | biopsy | 43-87 years | rectum | tubulovillous adenoma |  |  |  |
| HGD2_GSE37364 | biopsy |  | rectum | villous adenoma |  |  |  |
| HGD3_GSE37364 | biopsy |  | sigma | tubular adenoma |  |  |  |
| HGD4_GSE37364 | biopsy |  | rectum | tubulovillous adenoma |  |  |  |
| HGD5_GSE37364 | biopsy |  | sigma | tubular adenoma |  |  |  |
| HGD6_GSE37364 | biopsy |  | rectum | villous adenoma |  |  |  |
| HGD7_GSE37364 | biopsy |  | rectum | tubular adenoma |  |  |  |
| HGD8_GSE37364 | biopsy |  | colon asc | tubular adenoma |  |  |  |
| HGD9_GSE37364 | biopsy |  | sigma | tubular adenoma |  |  |  |
| HGD10_GSE37364 | biopsy |  | rectum | tubulovillous adenoma |  |  |  |
| HGD11_GSE37364 | biopsy |  | coecum | tubulovillous adenoma |  |  |  |
| HGD12_GSE37364 | biopsy |  | rectum | tubulovillous adenoma |  |  |  |
| HGD13_GSE37364 | biopsy |  | colon transv | villous adenoma |  |  |  |
| **CRC Dukes A, B** | |  |  | | | | |
| CRC1_GSE37364 | biopsy |  | rectum | adenocarcinoma | T1N0M0 | G2 | A |
| CRC2_GSE37364 | biopsy |  | colon asc | adenocarcinoma | T3N0M0 | G2 | B2 |
| CRC3_GSE37364 | biopsy |  | rectum | adenocarcinoma | T3N0M0 | G2 | B2 |
| CRC4_GSE37364 | biopsy |  | coecum | adenocarcinoma | T3N0M0 | G2 | B2 |
| CRC5_GSE37364 | biopsy |  | colon asc | adenocarcinoma | T3N0M0 | G2 | B2 |
| CRC6_GSE37364 | biopsy |  | coecum | adenocarcinoma | T2N0M0 | G2 | B1 |
| CRC7_GSE37364 | biopsy |  | coecum | adenocarcinoma | T3N0M0 | G2 | B2 |
| CRC8_GSE37364 | biopsy |  | colon transv. | adenocarcinoma | T3N0M0 | G2 | B2 |
| CRC9_GSE37364 | biopsy |  | rectum | adenocarcinoma | T2N0M0 | G1 | B1 |
| CRC10_GSE37364 | biopsy |  | rectum | adenocarcinoma | T3N0M0 | G1 | B2 |
| CRC11_GSE37364 | biopsy |  | sigma | adenocarcinoma | T3N0M0 | G2 | B2 |
| CRC12_GSE37364 | biopsy |  | colon asc. | adenocarcinoma | T2N0M0 | G1 | B1 |
| CRC13_GSE37364 | biopsy |  | colon asc. | adenocarcinoma | T3N0M0 | G2 | B2 |
| CRC14_GSE37364 | biopsy |  | flex.lienalis | adenocarcinoma | T3N0M0 | G1 | B2 |
| **CRC Dukes C, D** | | | | | | | |
| CRC15_GSE37364 | biopsy | 40-85 years | rectum-sigma | adenocarcinoma | T3N2M0 | G2 | C3 |
| CRC16_GSE37364 | biopsy |  | rectum | adenocarcinoma | T3N2M1 | G2 | D |
| CRC17_GSE37364 | biopsy |  | colon asc. | adenocarcinoma | T3N2M1 | G2 | D |
| CRC18_GSE37364 | biopsy |  | coecum | adenocarcinoma | T3N1M0 | G2 | C |
| CRC19_GSE37364 | biopsy |  | rectum | adenocarcinoma | T2N1M0 | G2 | C1 |
| CRC20_GSE37364 | biopsy |  | coecum | adenocarcinoma | T3N1M0 | G2 | C |
| CRC21_GSE37364 | biopsy |  | rectum | adenocarcinoma | T4N2M0 | G2 | C3 |
| CRC22_GSE37364 | biopsy |  | rectum | adenocarcinoma | T3N1M0 | G2 | C |
| CRC23_GSE37364 | biopsy |  | rectum | adenocarcinoma | T3N1M0 | G2 | C2 |
| CRC24_GSE37364 | biopsy |  | rectum | adenocarcinoma | T3N1M0 | G3 | C2 |
| CRC25_GSE37364 | biopsy |  | rectum | adenocarcinoma | T3N1M1 | G2 | D |
| CRC26_GSE37364 | biopsy |  | rectum | adenocarcinoma | T3N1M1 | G2 | D |
| CRC27_GSE37364 | biopsy |  | rectum | adenocarcinoma | T2N3M1 | G2 | D |

**GSE4183 – colorectal biopsy samples analyzed on HGU133Plus2.0 microarray**

| **Sample ID** | **Sample**  **type** | **Age range** | **Localization** | **Histology** | **TNM** | **Grade** | **Dukes** |
| --- | --- | --- | --- | --- | --- | --- | --- |
| **Normal** | | | | | | | |
| N1_GSE4183 | biopsy | 44-60 years |  | normal |  |  |  |
| N2_GSE4183 | biopsy |  |  | normal |  |  |  |
| N3_GSE4183 | biopsy |  |  | normal |  |  |  |
| N4_GSE4183 | biopsy |  |  | normal |  |  |  |
| N5_GSE4183 | biopsy |  |  | normal |  |  |  |
| N6_GSE4183 | biopsy |  |  | normal |  |  |  |
| N7_GSE4183 | biopsy |  |  | normal |  |  |  |
| N8_GSE4183 | biopsy |  |  | normal |  |  |  |
| **Low-grade dysplastic adenoma** | | | | | | | |
| LGD1_GSE4183 | biopsy | 37-83 years | rectum | tubulovillous adenoma |  |  |  |
| LGD2_GSE4183 | biopsy |  | coecum | tubulovillous adenoma |  |  |  |
| LGD3_GSE4183 | biopsy |  | sigma-colon desc | villous adenoma |  |  |  |
| LGD4_GSE4183 | biopsy |  | coecum | tubulovillous adenoma |  |  |  |
| LGD5_GSE4183 | biopsy |  | coecum | tubulovillous adenoma |  |  |  |
| LGD6_GSE4183 | biopsy |  | rectum | tubulovillous adenoma |  |  |  |
| LGD7_GSE4183 | biopsy |  | coecum | tubulovillous adenoma |  |  |  |
| LGD8_GSE4183 | biopsy |  | rectum | villous adenoma |  |  |  |
| LGD9_GSE4183 | biopsy |  | sigma | tubulovillous adenoma |  |  |  |
| **High-grade dysplastic adenoma** | | | | | | | |
| HGD1_GSE4183 | biopsy | 64-93 years | flexura hepatica | villous adenoma |  |  |  |
| HGD2_GSE4183 | biopsy |  | rectum | tubulovillous adenoma |  |  |  |
| HGD3_GSE4183 | biopsy |  | coecum | tubulovillous adenoma |  |  |  |
| HGD4_GSE4183 | biopsy |  | rectum | villous adenoma |  |  |  |
| HGD5_GSE4183 | biopsy |  | coecum | tubulovillous adenoma |  |  |  |
| HGD6_GSE4183 | biopsy |  | colon descendens | villous adenoma |  |  |  |
| **CRC** | | | | | | | |
| CRC1_GSE4183 | biopsy | 46-88 years | rectum | adenocarcinoma | T3N0M0 | G2 | B |
| CRC2_GSE4183 | biopsy |  | colon ascendens | adenocarcinoma | T3N0M0 | G3 | B |
| CRC3_GSE4183 | biopsy |  | hepatic flexure | adenocarcinoma | T2N0M0 | G1 | B |
| CRC4_GSE4183 | biopsy |  | sigma | adenocarcinoma | T3N0M0 | G2 | B |
| CRC5_GSE4183 | biopsy |  | colon descendens | adenocarcinoma | T4N0M0 | G1 | B |
| CRC6_GSE4183 | biopsy |  | rectum | adenocarcinoma | T2N0M0 | G2 | B |
| CRC7_GSE4183 | biopsy |  | coecum | adenocarcinoma | T3N0M0 | G2 | B |
| CRC8_GSE4183 | biopsy |  | colon descendens | adenocarcinoma | T2N1M0 | G2 | C |
| CRC9_GSE4183 | biopsy |  | flex. lienalis | adenocarcinoma | T3N1M1 | G2 | D |
| CRC10_GSE4183 | biopsy |  | sigma | adenocarcinoma | T4N1M0 | G1-2 | C |
| CRC11_GSE4183 | biopsy |  | sigma | adenocarcinoma | T4N0M1 | G1 | D |
| CRC12_GSE4183 | biopsy |  | rectum | adenocarcinoma | T3N1M1 | G1 | D |
| CRC13_GSE4183 | biopsy |  | rectum | adenocarcinoma | T3N0M1 | G1 | D |
| CRC14_GSE4183 | biopsy |  | coecum | adenocarcinoma | T3N1M1 | G2 | D |
| CRC15_GSE4183 | biopsy |  | rectum | adenocarcinoma | T2N0M1 | G1 | D |

**GSE10714 – colorectal biopsy samples analyzed on HGU133Plus2.0 microarray**

| **Sample ID** | **Sample**  **type** | **Age range** | **Localization** | **Histology** | **TNM** | **Grade** | **Dukes** |
| --- | --- | --- | --- | --- | --- | --- | --- |
| **Normal** | | | | | | | |
| N1_GSE10714 | biopsy | 44-60 years | - | normal |  |  |  |
| N2_GSE10714 | biopsy |  | - | normal |  |  |  |
| N3_GSE10714 | biopsy |  | colon asc. | normal |  |  |  |
| **Low-grade dysplastic adenoma** | | | | | | | |
| LGD1_GSE10714 | biopsy | 43-83 years | colon desc. | tubulovillous adenoma |  |  |  |
| LGD2_GSE10714 | biopsy |  | sigma | villous adenoma |  |  |  |
| LGD3_GSE10714 | biopsy |  | colon desc. | villous adenoma |  |  |  |
| **High-grade dysplasia** | | | | | | | |
| HGD1_GSE10714 | biopsy | 72-81 years | colon desc. | tubulovillous adenoma |  |  |  |
| HGD2_GSE10714 | biopsy |  | - | tubulovillous adenoma |  |  |  |
| **CRC** | | | | | | | |
| CRC1_GSE10714 | biopsy | 53-77 years | rectum | adenocarcinoma | T2N0M0 | B | G2 |
| CRC2_GSE10714 | biopsy |  | rectum | adenocarcinoma | T3N1M1 | D | G2 |
| CRC3_GSE10714 | biopsy |  | colon desc. | adenocarcinoma | T3N0M0 | B | G2 |
| CRC4_GSE10714 | biopsy |  | sigma | adenocarcinoma | T2N0M0 | B | G2 |
| CRC5_GSE10714 | biopsy |  | - | adenocarcinoma | T3N1M1 | D | G1 |
| CRC6_GSE10714 | biopsy |  | coecum | adenocarcinoma | T3N1M1 | D | G2 |
| CRC7_GSE10714 | biopsy |  | rectum | adenocarcinoma | T3N1M1 | D | G2 |

**GSE15960 – laser microdissected colorectal epithelial cells analyzed on HGU133Plus2.0 microarray**

| **Sample ID** | **Sample**  **type** | **Age range** | **Localization** | **Histology** | **TNM** | **Grade** | **Dukes** |
| --- | --- | --- | --- | --- | --- | --- | --- |
| **NAT** | | | | | | | |
| N1_GSE15960 | fresh frozen tissue | 66-79 years | sigma | NAT |  |  |  |
| N2_GSE15960 | fresh frozen tissue |  | rectum | NAT |  |  |  |
| N3_GSE15960 | fresh frozen tissue |  | sigma | NAT |  |  |  |
| N4_GSE15960 | fresh frozen tissue |  | rectum | NAT |  |  |  |
| N5_GSE15960 | fresh frozen tissue |  | colon descendens | NAT |  |  |  |
| N6_GSE15960 | fresh frozen tissue |  | colon descendens | NAT |  |  |  |
| **Adenoma** | | | | | | | |
| AD1_GSE15960 | fresh frozen tissue | 66-79 years | sigma | tubular adenoma |  |  |  |
| AD2_GSE15960 | fresh frozen tissue |  | rectum | tubular adenoma |  |  |  |
| AD3_GSE15960 | fresh frozen tissue |  | sigma | tubular adenoma |  |  |  |
| AD4_GSE15960 | fresh frozen tissue |  | rectum | tubular adenoma |  |  |  |
| AD5_GSE15960 | fresh frozen tissue |  | colon descendens | tubular adenoma |  |  |  |
| AD6_GSE15960 | fresh frozen tissue |  | colon descendens | tubular adenoma |  |  |  |
| **CRC** | | | | | | | |
| CRC1_GSE15960 | fresh frozen tissue | 66-79 years | sigma | adenocarcinoma | T2N0M0 | G2 | B |
| CRC2_GSE15960 | fresh frozen tissue |  | rectum | adenocarcinoma | T2N0M0 | G1 | B |
| CRC3_GSE15960 | fresh frozen tissue |  | sigma | adenocarcinoma | T3N0M0 | G2 | B |
| CRC4_GSE15960 | fresh frozen tissue |  | rectum | adenocarcinoma | T2N0M0 | G2 | B |
| CRC5_GSE15960 | fresh frozen tissue |  | colon descendens | adenocarcinoma | T3N0M0 | G2 | B |
| CRC6_GSE15960 | fresh frozen tissue |  | colon descendens | adenocarcinoma | T2N0M0 | G2 | B |
