# Supplementary material for: DNA hypermethylation and decreased mRNA expression of MAL, PRIMA1, PTGDR and SFRP1 in colorectal adenoma and cancer
Source: BMC Cancer. 2015 Oct 19;15:736. doi: 10.1186/s12885-015-1687-x (PMC4612409; doi:10.1186/s12885-015-1687-x)
Supplement: Additional file 2: Table S2. — Clinical data of patients involved in the study. (DOCX 24 kb) [file 12885_2015_1687_MOESM2_ESM.docx]

**Supplementary Table 2. Clinical data of the analyzed samples. Asterisk (*) indicates laser microdissected samples.**

| **Sample ID** | **Sample**  **type** | **Age range** | **Localization** | **Histology** | **TNM** | **Grade** | **Dukes** |
| --- | --- | --- | --- | --- | --- | --- | --- |
| **NAT** | | | | | | | |
| NAT1* | fresh frozen tissue | 60-91 years | sigma | NAT |  |  |  |
| NAT2* | fresh frozen tissue |  | rectum | NAT |  |  |  |
| NAT3* | fresh frozen tissue |  | colon descendens | NAT |  |  |  |
| NAT4* | fresh frozen tissue |  | sigma | NAT |  |  |  |
| NAT5* | fresh frozen tissue |  | colon ascendens | NAT |  |  |  |
| NAT6 | fresh frozen tissue |  | rectum | NAT |  |  |  |
| NAT7 | fresh frozen tissue |  | coecum | NAT |  |  |  |
| NAT8 | fresh frozen tissue |  | coecum | NAT |  |  |  |
| NAT9 | fresh frozen tissue |  | sigma | NAT |  |  |  |
| NAT10 | fresh frozen tissue |  | sigma | NAT |  |  |  |
| NAT11 | fresh frozen tissue |  | colon ascendens | NAT |  |  |  |
| NAT12 | fresh frozen tissue |  | colon ascendens | NAT |  |  |  |
| NAT13 | fresh frozen tissue |  | coecum | NAT |  |  |  |
| NAT14 | fresh frozen tissue |  | sigma | NAT |  |  |  |
| NAT15 | fresh frozen tissue |  | colon ascendens | NAT |  |  |  |
| **Adenoma** | | | | | | | |
| Ad1* | fresh frozen tissue | 48-78 years | rectum | tubular adenoma |  |  |  |
| Ad2* | fresh frozen tissue |  | rectum | tubular adenoma |  |  |  |
| Ad3* | fresh frozen tissue |  | sigma | tubular adenoma |  |  |  |
| Ad4* | fresh frozen tissue |  | colon descendens | tubular adenoma |  |  |  |
| Ad5* | fresh frozen tissue |  | colon descendens | tubular adenoma |  |  |  |
| Ad6 | biopsy |  | sigma | tubular adenoma |  |  |  |
| Ad7 | biopsy |  | sigma | tubular adenoma |  |  |  |
| Ad8 | biopsy |  | coecum | tubular adenoma |  |  |  |
| Ad9 | biopsy |  | colon descendens | tubular adenoma |  |  |  |
| Ad10 | biopsy |  | sigma | tubular adenoma |  |  |  |
| Ad11 | biopsy |  | flexura hepatica | tubulovillous adenoma |  |  |  |
| Ad12 | biopsy |  | coecum | tubulovillous adenoma |  |  |  |
| Ad13 | biopsy |  | sigma | tubulovillous adenoma |  |  |  |
| Ad14 | biopsy |  | colon descendens | tubulovillous adenoma |  |  |  |
| Ad15 | biopsy |  | rectum | tubulovillous adenoma |  |  |  |
| **CRC** | | | | | | | |
| CRC1* | fresh frozen tissue | 60-91 years | sigma | adenocarcinoma | T3N0M0 | G2 | B |
| CRC2* | fresh frozen tissue |  | rectum | adenocarcinoma | T3N0M0 | G2 | B |
| CRC3* | fresh frozen tissue |  | colon descendens | adenocarcinoma | T2N0M0 | G2 | B |
| CRC4* | fresh frozen tissue |  | sigma | adenocarcinoma | T3N0M0 | G2 | B |
| CRC5* | fresh frozen tissue |  | colon ascendens | adenocarcinoma | T3N0M0 | G2 | B |
| CRC6 | fresh frozen tissue |  | rectum | adenocarcinoma | T3N2M0 | G1 | C |
| CRC7 | fresh frozen tissue |  | coecum | adenocarcinoma | T3N0M0 | G2 | B |
| CRC8 | fresh frozen tissue |  | coecum | adenocarcinoma | T3N0M0 | G3 | B |
| CRC9 | fresh frozen tissue |  | sigma | adenocarcinoma | T3N1M0 | G2 | B |
| CRC10 | fresh frozen tissue |  | sigma | adenocarcinoma | T3N2M0 | G2 | C |
| CRC11 | fresh frozen tissue |  | colon ascendens | adenocarcinoma | T3N2M0 | G2 | C |
| CRC12 | fresh frozen tissue |  | colon ascendens | adenocarcinoma | T3N2M0 | G2 | C |
| CRC13 | fresh frozen tissue |  | coecum | adenocarcinoma | T3N2M0 | G1 | C |
| CRC14 | fresh frozen tissue |  | sigma | adenocarcinoma | T3N0M0 | G1 | B |
| CRC15 | fresh frozen tissue |  | colon ascendens | adenocarcinoma | T3N1M0 | G2 | C |
