# Supplementary figures and images for: DNA hypermethylation and decreased mRNA expression of MAL, PRIMA1, PTGDR and SFRP1 in colorectal adenoma and cancer
Source: BMC Cancer. 2015 Oct 19;15:736. doi: 10.1186/s12885-015-1687-x (PMC4612409; doi:10.1186/s12885-015-1687-x)

Supplementary Fig 1

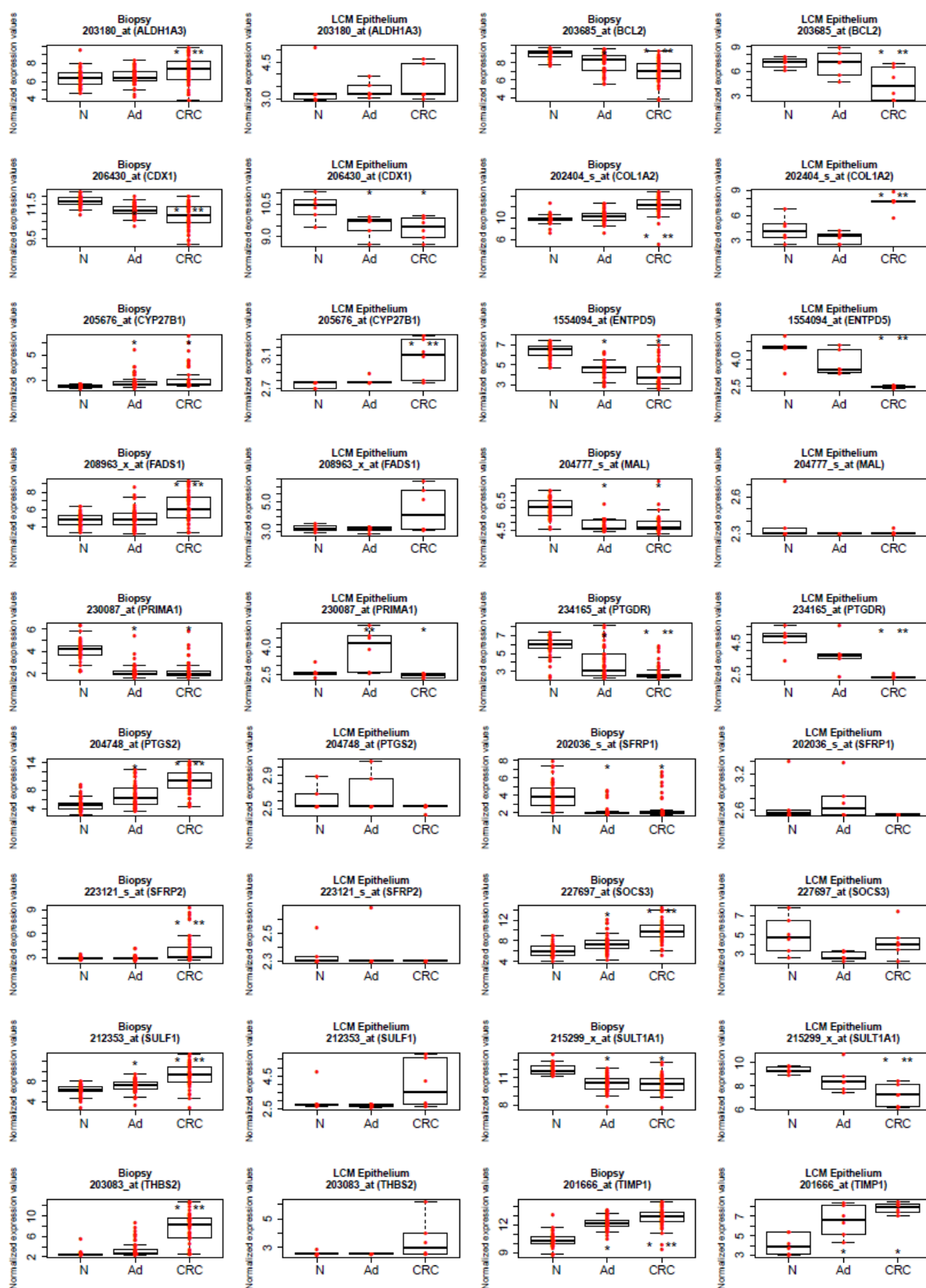

Supplement: Additional file 3: Figure S1. — Gene expression of the selected marker set in normal (n = 49), adenoma (n = 49), and tumour (n = 49) biopsy samples. Results are represented as pairwise box plots showing single gene expession in the biopsy and in the laser microdissected epithelial cells from normal (n = 6), adenoma (n = 6), and tumour (n = 6). Red dots are normalised gene expression values, box plots represent median and standard deviation of the data. Asterisk (*) represents significance (p < 0.05) in adenoma and tumour samples compared to normals and double asterisk (**) represents significance (p < 0.05) in tumour samples compared to adenomas. Each box plot has individual scale of gene expression. Changes in the direction of gene expression was found to be similar in colon biopsies and in LCM epithelial cells. (PDF 296 kb) [file 12885_2015_1687_MOESM3_ESM.pdf]

Supplementary Fig 2

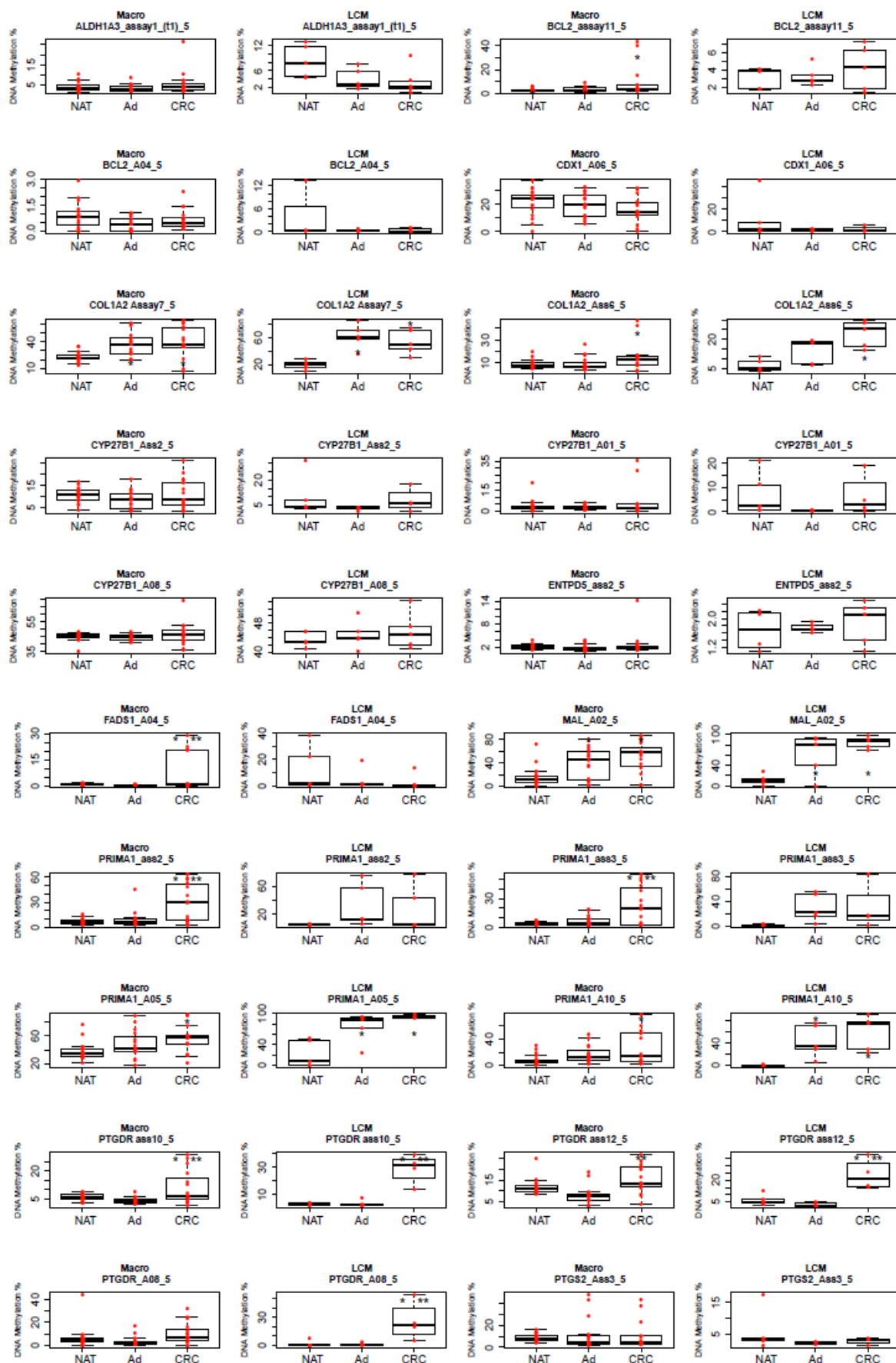

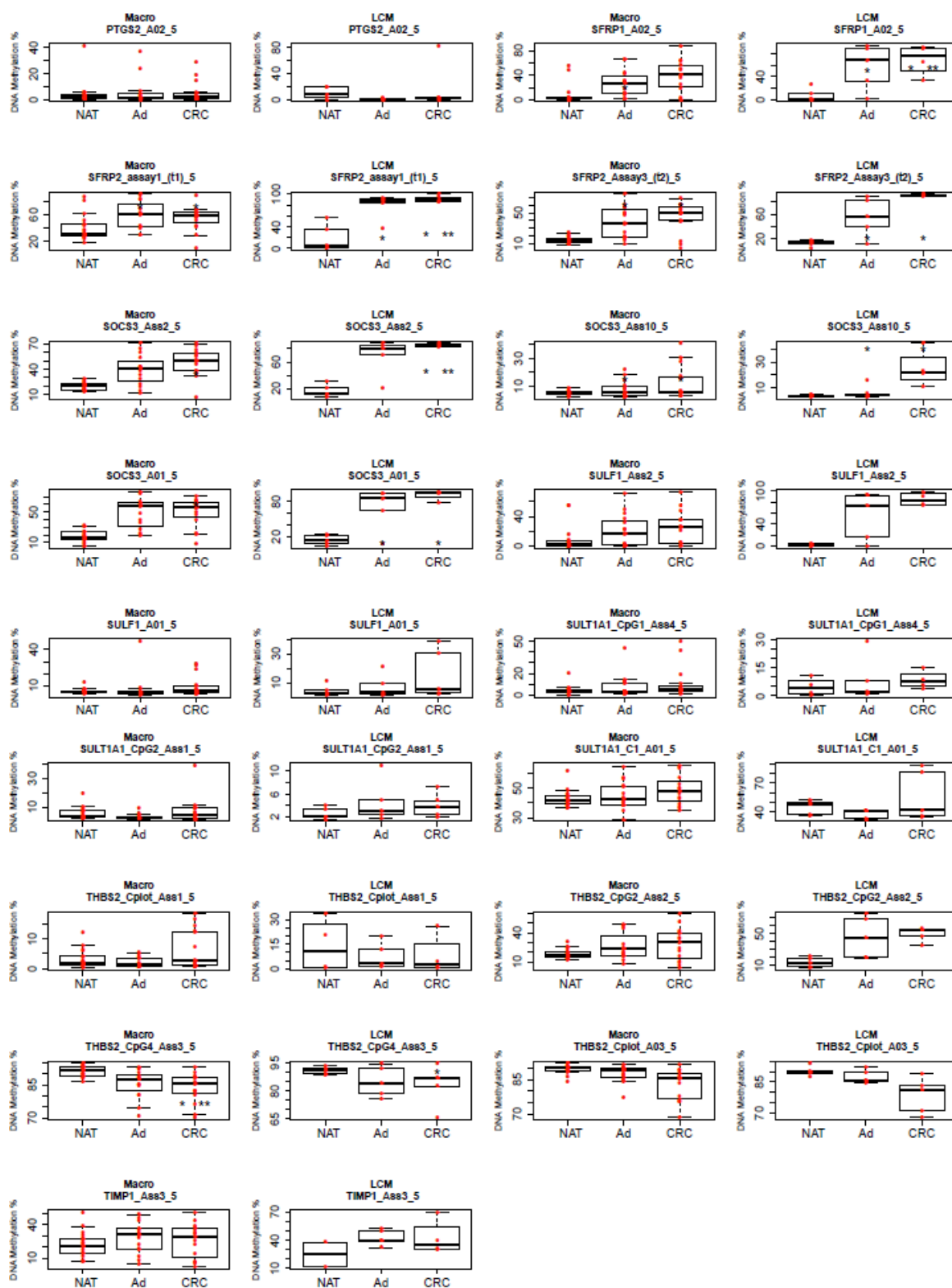

Supplement: Additional file 5: Figure S2. — Box plot representation of DNA methylation alterations of analysed genes in biopsies and macrodissected specimens of NAT (n = 10), AD (n = 10), and CRC (n = 10) and in LCM epithelial cells of NAT (n = 5), AD (n = 5) and CRC (n = 5). Red dots are individual DNA methylation percent values, box plots represent median and standard deviation of the data. Asterisk (*) represents significance (p < 0.05) in adenoma and tumour samples compared to normals and double asterisk (**) represents significance (p < 0.05) in tumour samples compared to adenomas. Each box plot has individual scale of DNA methylation percent. Certain genes showe hypermethylation in colon biopsies and also in LCM epithelial cells (e.g. MAL, PRIMA1, PTGDR, SFRP2), while there were genes with no remarkable DNA methylation level alterations (e.g. BCL2, CDX1, PTGS2, SULF1). Macro = macrodissected samples, LCM = laser microdissected epithelial cells. (PDF 359 kb) [file 12885_2015_1687_MOESM5_ESM.pdf]
